# Fostering Refugee and Other Migrant Resilience through Empowerment, Pluralism, and Collaboration in Mental Health

**DOI:** 10.3390/ijerph17249557

**Published:** 2020-12-21

**Authors:** Azaad Kassam, Olivia Magwood, Kevin Pottie

**Affiliations:** 1 Department of Psychiatry, University of Ottawa, 75 Laurier Ave. E, Ottawa, ON K1N 6N5, Canada; 2 Pinecrest-Queensway Community Health Centre, 1365 Richmond Rd #2, Ottawa, ON K2B 6R7, Canada; 3 Ottawa Newcomer Health Centre, 291 Argyle, Ottawa, ON K2P 1B8, Canada; 4 C.T. Lamont Primary Care Research Centre, Bruyère Research Institute, 85 Primrose Ave, Ottawa, ON K1R 6M1, Canada; omagwood@bruyere.org (O.M.); kpottie@uottawa.ca (K.P.); 5 Interdisciplinary School of Health Sciences, Faculty of Health Sciences, University of Ottawa, 75 Laurier Ave. E, Ottawa, ON K1N 6N5, Canada; 6 Department of Family Medicine, University of Ottawa, 75 Laurier Ave. E, Ottawa, ON K1N 6N5, Canada; 7 School of Epidemiology and Public Health, University of Ottawa, 75 Laurier Ave. E, Ottawa, ON K1N 6N5, Canada

“Although the world is full of suffering, it is also full of the overcoming of it.

—Helen Keller

Suffering is a part of the human experience [1], but many suffer in unimaginable ways at the hands of fellow human beings. War, indeed, is the most serious threat to human health. What is lost in war? Our possessions, means, land, institutions, and schools; our loved ones, sense of security, and culture; our freedom, peace of mind, and dignity. For many, war also forces people to abandon their homeland in search of safety and a future. War destroys communities and families and disrupts the development of the social and economic fabric of nations. The result is poverty, the fragmentation of society, and suffering.

The trauma of war and conflict has a major impact on the mind and body. It changes the structure of our brain and shifts the way we think, feel, and perceive our world. Torture, loss, and separation often lead to mental illness. Refugees and asylum seekers have high and persistent rates of PTSD (post-traumatic stress disorder) and depression [2] among other mental health concerns. It is tempting to pathologize the effects of war. Perhaps we do this to better comprehend the unfathomable horrors our patients have experienced and their ensuing strife. Psychiatric diagnoses become our attempt to structure senseless events. However, the psychological fallout of war is more complex than a psychiatric diagnosis [3].

Health care providers can meaningfully integrate some of this complexity within the context of global mental health. Research involving displaced persons is increasingly focused on helping those affected by the trauma of war. Global and public health principles emphasize the central importance of social determinants of health. Evidence suggests that it is not the trauma of war but rather post-migratory factors, such as the discrimination that accompanies resettlement, that have the greatest health impacts [4,5].

Systemic racism is a significant determinant of mental health [5]. Discrimination has long plagued migrant populations, often beginning in the country of origin, in transit, and commonly in the resettlement country. Refugees are often victims of xenophobia—fears that newcomers will steal jobs and negatively affect the host society. In contrast, research demonstrates that refugees can positively influence the economic growth and cultural enrichment of the receiving country [6,7,8].

Stigma often accompanies the “refugee” and “migrant” labels, exacerbating the distress of those who have already experienced significant hardship. We often put refugees in a position of vulnerability, expecting them to be weak so that we can lift them up. We expect their gratitude for being allowed to live in our country, amongst us, based on that expectation of vulnerability [4]. We injure their humanity each time we fail to privilege their voices. Evidence-based guidelines recognize these harms [9]. Refugee patients will sometimes share their suffering with care providers they trust. We sometimes forget that the most uplifting thing we can do for someone is to empower them, to facilitate their sense of agency. We need to bring services to communities where people break bread, raise families, and live their lives. Interventions that improve mental health outcomes include supporting people’s cultural, religious, and spiritual practices, supporting caregivers, keeping families intact, stabilizing housing, and improving access to education and employment, as well as primary health care.

Most refugees experience normal responses to abnormal levels of stress. However, there are also many who can benefit from the treatment of mental health conditions, provided they have access to services [10]. For many reasons, specialized clinical settings, such as hospitals, are limited in what they do for newcomers. Language, fears of the unknown and unfamiliar, and practical barriers—getting time off work, having no one to care for children, and having no money to travel—all prevent refugees from accessing services. Community-based services should be developed. Bringing together primary care practitioners with mental health professionals can improve accessibility. A variety of clinical interventions and settings [11] have had success, especially creative arts therapies—drama, music, dance, and visual arts. Talk therapy remains an important method, but there is no universally applicable psychotherapy. The concept of talking about one’s trauma is based on a Western psychological paradigm. With the right person, at the right time, in the right context, culturally safe talk therapy can be life-changing, and even life-saving. Narrative exposure therapy (NET), for example, has been used successfully with many cultural groups, across ages and genders [10]. It integrates coherent life stories, recognizing the negative experiences and helping to find meaningful, positive memories.

In community settings, it is critical to work with culture. Clinical tools such as the Cultural Formulation can guide our assessment [12]. This includes listening to how people experience their suffering, how they understand their predicament, what words they use to describe their distress, and what their strengths are. Humility and a pluralistic mindset are essential. We often refer to what we “know” and what others “believe”. What makes one culture’s way of understanding distress superior to another’s? If we allow different realities to co-exist, we might learn something novel that inspires a more creative approach to helping the person in front of us.

One woman witnessed the death of her 10-year-old child in an explosion in the Syrian war. She then found herself floating on a capsized boat in the Mediterranean and watched another child drown, unable to help. In the resettlement country, she remained haunted by these events and presented to her doctor with symptoms of trauma. Remarkably, however, she remained hopeful about the future by seeking to make meaning of these experiences and felt welcomed by the host country. The perspective this woman had developed in the face of adversity highlights her resilience. If you have belonging, meaning, purpose, and hope [13], perhaps you can survive anything.

In summary, to assist refugees and other migrants, we must work together to help people in the community access culturally safe mental health services in the primary care setting. Empowerment of refugees fosters resilience. After all, despite the atrocities and assaults on human dignity inflicted by war, refugees reveal to us the human capacity to survive and thrive.

## References

[1] Kleinman A. (1995). Writing at the Margin: Discourse between Anthropology and Medicine.

[2] Blackmore R., Boyle J.A., Fazel M., Ranasinha S., Gray K.M., Fitzgerald G., Misso M., Gibson-Helm M. (2020). The prevalence of mental illness in refugees and asylum seekers: A systematic review and meta-analysis. PLoS Med..

[3] Roupetz S., Bartels S.A., Michael S., Najjarnejad N., Anderson K., Davison C. (2020). Displacement and Emotional Well-Being among Married and Unmarried Syrian Adolescent Girls in Lebanon: An Analysis of Narratives. Int. J. Environ. Res. Public Health.

[4] Kirmayer L.J., Ryder A.G. (2016). Culture and psychopathology. Curr. Opin. Psychol..

[5] Rousseau C. (2018). Addressing Mental Health Needs of Refugees. Can. J. Psychiatry.

[6] Betts D.A., Bloom L., Kaplan D.J., Omata D.N. (2014). Refugee Economies: Rethinking Popular Assumptions.

[7] Miller S.D. (2018). Assessing the Impacts of Hosting Refugees.

[8] Beiser M. (2009). Resettling refugees and safeguarding their mental health: Lessons learned from the Canadian Refugee Resettlement Project. Transcult. Psychiatry.

[9] Pottie K., Greenaway C., Feightner J., Welch V., Swinkels H., Rashid M., Narasiah L., Kirmayer L.J., Ueffing E., MacDonald N.E. (2011). Evidence-based clinical guidelines for immigrants and refugees. CMAJ.

[10] Gruner D., Magwood O., Bair L., Duff L., Adel S., Pottie K. (2020). Understanding Supporting and Hindering Factors in Community-Based Psychotherapy for Refugees: A Realist-Informed Systematic Review. Int. J. Environ. Res. Public Health.

[11] Lu J., Jamani S., Benjamen J., Agbata E., Magwood O., Pottie K. (2020). Global Mental Health and Services for Migrants in Primary Care Settings in High-Income Countries: A Scoping Review. Int. J. Environ. Res. Public Health.

[12] Kirmayer L.J., Fung K., Rousseau C., Lo H.T., Menzies P., Guzder J., Ganesan S., Andermann L., McKenzie K. (2020). Guidelines for Training in Cultural Psychiatry. Can. J. Psychiatry.

[13] Restoule B.M., Hopkins C., Robinson J., Wiebe P.K. (2016). First Nations Mental Wellness: Mobilizing Change through Partnership and Collaboration. Can. J. Community Ment. Health.

